# Supplementary material for: The Use of Biosilica to Increase the Compressive Strength of Cement Mortar: The Effect of the Mixing Method
Source: Materials (Basel). 2023 Aug 8;16(16):5516. doi: 10.3390/ma16165516 (PMC10456586; doi:10.3390/ma16165516)
Supplement: Supplementary file 1 [file materials-16-05516-s001.zip › materials-2453207-SI.pdf]

materials

MDPI

Supplementary Material

Figure S1a,b show the flexural strength of cement mortars with different wt.% of biosilica for 7 and 28 days, respectively. B0, B1, B2, B3 and B4 correspond to the 0%, 2.5%, 5%, 7.5% and 10% of biosilica respectively. Presented results, are obtained when biosilica was mixed with cement (mixing method 1).

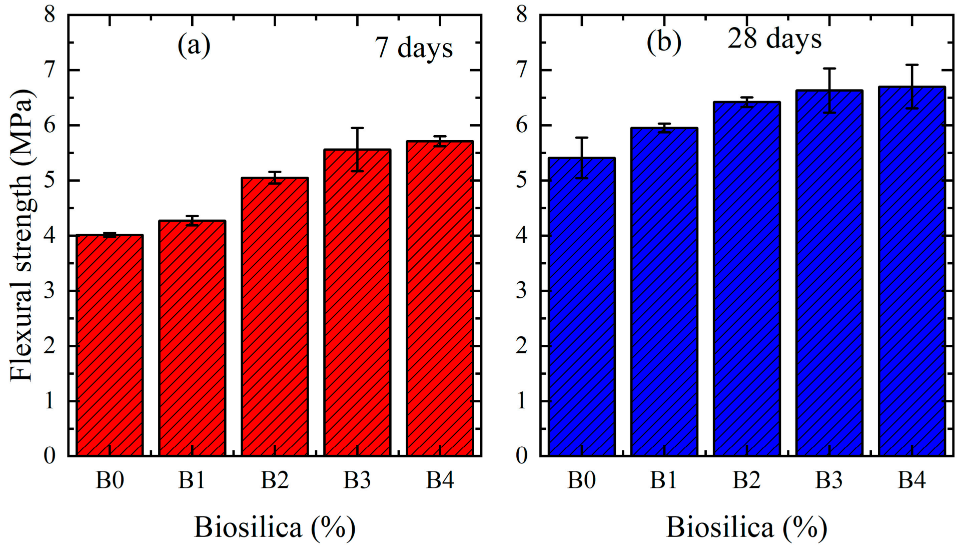

Figure S1. Flexural strength of cement mortars with different wt.% of biosilica. The results given for the mixing method 1.

Figure S2a,b show the flexural strength of cement mortars with different wt.% of biosilica for 7 and 28 days, respectively. These results, are obtained when biosilica was mixed with water (mixing method 2).

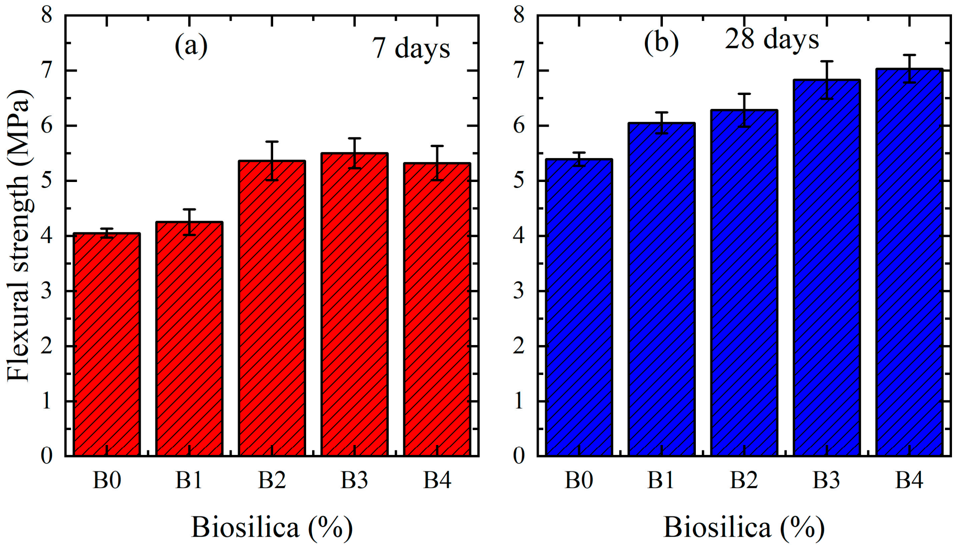

Figure S2. Flexural strength of cement mortars with different wt.% of biosilica. The results given for the mixing method 2.
